# Toward human-level concept learning: Pattern benchmarking for AI algorithms

**DOI:** 10.1016/j.patter.2023.100788

**Published:** 2023-07-05

**Authors:** Andreas Holzinger, Anna Saranti, Alessa Angerschmid, Bettina Finzel, Ute Schmid, Heimo Mueller

**Affiliations:** 1 Human-Centered AI Lab, University of Natural Resources & Life Sciences Vienna, Vienna, Austria; 2 University of Bamberg, Bamberg, Germany; 3 Medical University Graz, Graz, Austria

**Keywords:** artificial intelligence, concept learning, pattern analysis, diagnostic datasets, benchmarks

## Abstract

Artificial intelligence (AI) today is very successful at standard pattern-recognition tasks due to the availability of large amounts of data and advances in statistical data-driven machine learning. However, there is still a large gap between AI pattern recognition and human-level concept learning. Humans can learn amazingly well even under uncertainty from just a few examples and are capable of generalizing these concepts to solve new conceptual problems. The growing interest in explainable machine intelligence requires experimental environments and diagnostic/benchmark datasets to analyze existing approaches and drive progress in pattern analysis and machine intelligence. In this paper, we provide an overview of current AI solutions for benchmarking concept learning, reasoning, and generalization; discuss the state-of-the-art of existing diagnostic/benchmark datasets (such as CLEVR, CLEVRER, CLOSURE, CURI, Bongard-LOGO, V-PROM, RAVEN, Kandinsky Patterns, CLEVR-Humans, CLEVRER-Humans, and their extension containing human language); and provide an outlook of some future research directions in this exciting research domain.

## The bigger picture

Due to great advances in statistical data-driven machine learning and the large amounts of data available for this purpose today, artificial intelligence (AI) applications have been very successful in standard pattern-recognition tasks. However, there is still a large gap between AI pattern recognition and human-level concept learning. Humans are surprisingly good at learning from just a few examples, even under uncertainty, and are able to generalize these concepts to solve new previously unknown conceptual problems. For example, it is very easy for humans to recognize concepts such as right/left, up/down, and big/small, which is a big challenge for machines. The international AI community is continuously developing new experimental environments and diagnostic/benchmark datasets to support future developments in the field of concept learning.

## Graphical abstract

**Figure:**
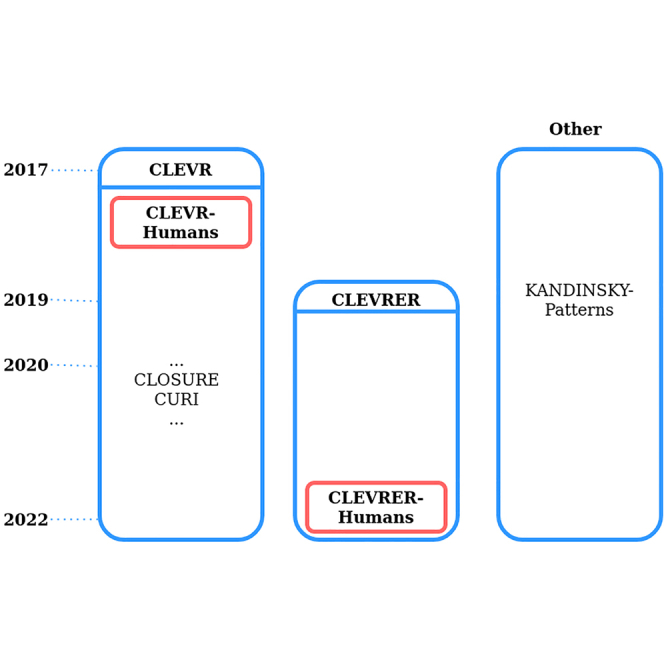

## Introduction

Artificial intelligence (AI) is making significant progress in a wide range of applications due to the availability of large amounts of data and the great success of data-driven statistical machine learning. Examples of AI applications for patterns include the following:

Computer vision: AI algorithms are being used to analyze and recognize patterns in images and video, allowing them to perform tasks such as image classification, object detection, and facial recognition.^2^

Predictive analytics: AI algorithms are being used to analyze patterns in data to make predictions about future events or outcomes. This can be used in applications such as stock market forecasting, fraud detection, and customer churn prediction.^3^

Robotics: AI algorithms are being used to enable robots to recognize and respond to patterns in their environment, allowing them to perform tasks such as navigating through unknown environments, handling and manipulating objects, and interacting with humans.^4^

Natural language processing (NLP): AI algorithms are being used to understand and generate human language,^5^ allowing them to perform tasks such as language translation, text summarization, and sentiment analysis.^6^

Natural language understanding (NLU): is a key component of NLP and includes the ability of AI to understand and interpret human language in a way that is similar to how a human would understand it, and is used, e.g., in chat-bots (e.g., ChatGPT^7^).

There is no debate that the trend toward using AI will continue to increase in the future. AI technology has the potential to improve efficiency, performance, and productivity in a wide range of applications and there is increasing research and development in this field. There are also increasing efforts to make AI more transparent, accountable, interpretable, explainable, and robust. Robustness and explainability will likely lead to greater trust^8^ in and adoption of trustworthy AI technology, particularly in domains that affect human life, for example in medicine/health^9^ or agriculture/forestry.^10^

Pattern recognition, as the process of identifying patterns in data and using those patterns to make predictions and/or decisions, is a central part of machine learning, and therefore a key aspect of AI becoming increasingly important in the future. Recent examples have demonstrated that AI can reach human-level performance and beyond,^11^ even in complex domains such as medicine.^12^^,^^13^^,^^14^

However, AI models are highly dependent on the quality of the input data and especially on the training data. Thus, it is essential that researchers understand the datasets and how they might affect the system’s performance.^15^ Although the latter is of eminent importance for learning, they are typically treated as pre-defined, static information; i.e., the current best models are passive and rely on human-curated training data but have no control over themselves. This is in contrast to how humans learn, because humans interact with their environment to gain information.^16^ The role of interactivity, which is especially important for learning new concepts, and the extent to which the learner can take an active role in learning those concepts have been considered extremely important by the AI research community for a very long time.^17^ Unlike AI, sometimes—of course, not always—humans are very good at understanding and explaining concepts, even in novel situations with complex dynamics and even with little interaction.^18^

Human conceptual abilities are also very productive: humans can understand and generate novel concepts through compositions of existing concepts, unlike standard machine classifiers, which are limited to a fixed set of classes. Moreover, humans are able to induce “*ad hoc*” categories.^19^ Thus, unlike AI systems, humans reason seamlessly in large, essentially “unbounded” concept spaces and are very good at dealing with uncertainty and under-determination.^20^ Overall, AI systems are designed to perform specific tasks or functions more efficiently or accurately than humans, but they do not have the same broad range of abilities or characteristics as humans.

Human intelligence and AI are two forms of intelligence that are different in many ways. Human intelligence refers to the mental abilities of people, such as the ability to perceive, learn, reason, adapt to new situations, and solve problems. Intelligence is a complex and multifaceted concept that encompasses many different cognitive and emotional capabilities. AI, on the other hand, refers to the ability of a machine or computer system to perform tasks that would normally require human intelligence, such as learning, problem solving, and decision making. AI can be trained to perform a wide range of tasks using prior knowledge, algorithms, and data, and it can adapt and improve its performance over time through machine learning.

This paper is organized as follows. After the introduction, in which we motivate the need for AI while highlighting the advantages of humans, in section ”background,” we explain some differences between human intelligence and artificial intelligence, human learning and machine learning in general, and human concept learning and machine concept learning in particular, and provide a very brief introduction to human visual information processing. In section “synthetic datasets for benchmarking concept learning, reasoning, and generalization,” we provide an overview of synthetic datasets for benchmarking concept learning, reasoning, and generalization. In section “AI solutions,” we present AI solutions that build on the datasets discussed in section “synthetic datasets for benchmarking concept learning, reasoning, and generalization.” Finally, section “future challenges and research directions” presents some selected future challenges and future research directions, and in section “conclusion” we have a short conclusion.

## Background

There are several key differences between human intelligence and AI (there are many good references, e.g., Hernández-Orallo and Chollet^21^^,^^22^).

Origin: human intelligence is a natural and inherent capability of humans, while AI is an artificial construct created by humans.

Scope: human intelligence is broad and multifaceted, encompassing many different mental abilities and functions, while AI is more specialized and focused on specific tasks or domains.

Learning: human intelligence is primarily developed through experience and learning, while AI can be trained and fine-tuned using algorithms and data.

Creativity: human intelligence is capable of creative thought and expression, while AI is limited to the capabilities and knowledge that it has been programmed or trained with.

Overall, human intelligence and AI are two different forms of intelligence that have their own unique capabilities and limitations. Although still difficult, it is nevertheless easier to define human and especially machine learning; the latter, especially statistical data-driven machine learning, has a very clear textbook definition. Human learning and machine learning are two different processes that are used to acquire knowledge and skills.

Human learning is the process by which humans acquire knowledge and skills through experience, education, and training. It is a complex and multifaceted process that involves various cognitive and emotional capabilities, such as perception, memory, problem-solving, and decision making.

Machine learning, on the other hand, is a clearly defined subfield of AI that involves the development of algorithms that can learn from data and improve their performance over time. Machine learning algorithms are designed to recognize patterns in data and use those patterns to make predictions or decisions. There are several key differences between human learning and machine learning:

Origin: human learning is a natural process that occurs in humans, while machine learning is an artificial construct created by humans.

Learning process: human learning involves complex cognitive and emotional processes, while machine learning involves the use of algorithms and data to learn and adapt.

Scale: human learning is a relatively slow process compared with machine learning, which can process and learn from large amounts of data very quickly.

Adaptability: human learning is highly adaptable and can learn in a wide range of contexts and environments, while machine learning is limited to the capabilities and knowledge that it has been programmed or trained with.

Human concept learning and machine concept learning are two different processes that are used to acquire and represent knowledge about a concept.

Human concept learning is the process by which humans acquire and represent knowledge about a concept.^23^ Such a concept is a mental representation of a category or an idea that allows the individual person to identify, classify, and understand objects, events, experiences, etc. Consequently, concept learning is a complex and multifaceted process that involves various cognitive and emotional capabilities, such as perception, memory, problem-solving, and decision making.^24^ Humans are able to learn concepts in a wide range of contexts and environments, and they can adapt and modify their understanding of concepts over time. Human concepts can be abstract or concrete, and they can be defined in a variety of ways, depending on the context in which they are used.^25^ For example, the concept of “dog” is an abstract concept that is used to represent a category of certain animals, while the concept of “red” is a concrete concept that is used to represent a specific color. Such concepts are formed through experience and learning, and they play a central role in the way that people think, communicate, and interact with the world. They allow individuals to make sense of their environments and to understand and communicate complex ideas within the social world.

Machine concept learning, on the other hand, is the process of acquiring and representing knowledge about a concept using machine learning algorithms and data. Machine concept learning involves the use of algorithms to recognize patterns in data and use those patterns to make predictions or decisions. Machine concept learning is limited to the capabilities and knowledge that have been programmed or trained into the algorithms and is not as adaptable as human concept learning.^26^ In contrast, human visual information processing involves several phases, including the following:

Sensory input: when light enters the eye, it is focused onto the retina, which is a layer of cells at the back of the eye that contains photoreceptors. These photoreceptors convert the light into electrical signals that are transmitted to the brain.^27^

Early processing: the electrical signals from the retina are transmitted to the primary visual cortex, which is a region of the brain located in the occipital lobe. The primary visual cortex performs the initial processing of visual information, including simple tasks such as edge detection and basic object recognition.^28^

Higher-level processing: the primary visual cortex sends the processed visual information to other regions of the brain for further processing. These regions include the inferior temporal cortex, which is involved in more complex tasks such as object recognition and face recognition, and the parietal lobe, which is involved in spatial attention and eye movements.^29^

Integration with other senses: the processed visual information is also integrated with information from other senses, such as hearing, touch, and proprioception (the sense of body position and movement). This integration allows us to build a more complete and coherent representation of the world around us.^30^

Overall, the human visual system is a sophisticated system that allows us to interpret and make sense of the visual information that humans receive from the world around. One of the most important aspects is compositionality. The expressionist artist Wassily Kandinsky promoted simple colors and simple shapes and in 1926 published his book, Point and Line to Plain,” a contribution to the analysis of painting elements.^31^ In 1959, Hubel and Wiesel^32^ carried out their famous experiments where they discovered that the visual system of the cat brain builds up an image from very simple elements into more complex representations. Humans group segments into objects and use concepts of object permanence and object continuity to explain what has happened and infer what will happen, and also to imagine what would happen in counterfactual situations. The problem of complex visual understanding has long been studied in computer vision.^33^ This later inspired the deep-learning pioneers to use this compositionality in neural networks. A deep-learning architecture can be viewed as a multilayer stack of simple modules, most of which are subject to learning and many of which compute non-linear input-output mappings. Each module in the stack transforms its input to increase both the selectivity and invariance of a representation. With multiple such non-linear layers, a system can implement complicated functions of its inputs that are simultaneously sensitive to detail and insensitive to large irrelevant variations such as background, pose, lighting, and surrounding objects. At first, edges and lines are learned, then shapes, and then objects are formed, eventually leading to concept representations.^34^

## Synthetic datasets for benchmarking concept learning, reasoning, and generalization

### The path from visual question-answering systems to corresponding benchmark datasets

One of the first datasets that were used for image classification tasks was Common Objects in Context (COCO).^35^^,^^36^ It contained images of objects mostly in their natural surroundings. Machine learning models were challenged with the tasks of object localization as well as the prediction of semantic descriptions (captions) of the content. One of the earliest works that learn captions from data does not use neural networks but conditional random fields (CRFs).^37^ In the case where neural networks are used, the general architecture to solve this task contains a convolutional neural network (CNN) that processes the image and extracts feature embeddings (usually corresponding to particular regions) that will be used as input to a bidirectional long short-term memory (biLSTM) network that aligns those visual embeddings with the embeddings of the caption.^38^^,^^39^ The prediction of the semantic description is based on the correlation of the image and with a set of possible captions, which are considered weak labels. Further research demonstrated the role of the image context (surroundings) in the performance of those methods,^40^ especially for the objects are relatively small compared with the others as well as partially occluded by others. Explainable AI (xAI) methods, such as layer-wise relevance propagation (LRP)^41^ and first-order interpretability logic (FOIL)^42^ also showed how artifacts in datasets can be misused by those models.^43^

One of the first visual question-answering datasets is called visual question answering (VQA and was created in 2015.^33^ The dataset consisted of real images as well as questions that were created based on human-generated captions. State-of-the-art architectures of that time provided good performance, but their generalization and compositionality were questioned and tested with an extension of the original dataset called Compositional VQA (C_VQA).^44^ This dataset was carefully crafted so that the distribution of questions across splits (compared with its first version^33^) remains the same across splits, but the answer distributions for a particular question type should be different. All architectures showed a drop in performance on the new dataset, even the Neural Module Networks (NMNs)^45^ (see section “NMNs”), which have a built-in compositional architecture. This is assumed to be because of the long short-term memory (LSTM) that uses strong language priors, which, in the case of the original dataset, are similar for training and test sets, but in C_VQA this was no longer the case.

After the captioning tasks reached a desirable performance with neural networks, one research direction proceeded into question-answering (QA) systems, where corresponding datasets such as the Visual Genome^46^ also evolved. Instead of hard or soft labeling, it was relevant to find out if neural networks are capable of answering user-posed questions about some properties of objects in the image. For an AI system to support human dialogue is more natural and more informative, since some information is encoded in the question,^47^ but for a neural network to be able to answer correctly a set of questions, following some pre-specified structure and usually increasing complexity, means that it can handle concept learning as well as having reasoning abilities, which go beyond embedding alignment. In general, questions and answers in future research will depart from template-generated constrained versions, will be longer, will contain more combinations of objects, concepts, and ideally will be free text.^44^ The same applies to images that depict more real-life elements than carefully constructed rendered scenes as well as videos. Currently, video QA systems (VideoQA) are evolving, with the laborious task of gathering appropriate data.^48^ An overview of VQA systems, their properties, and abilities is provided by Kojima et al.^49^

Benchmarking the performance of complex VQA systems led to the programmatical creation of the Compositional Language and Elementary Visual Reasoning diagnostics dataset (CLEVR)^50^ in 2017, which is analyzed in section “CLEVR.” This was the first research work presenting a synthetic dataset that stated clearly the necessity of quantifying some preliminary capabilities of compositionality and generalization of AI solutions. The inventors did not just create the dataset and the corresponding reasoning challenges, and provided an AI algorithm that managed to tackle them to a certain degree.

After the publication of CLEVR, there was a plethora of extensions of this dataset (CLEVR_HYP, Sort-of-CLEVR, Quantificational Language and Elementary Visual Reasoning [QLEVR], CLEVR-XAI, Super-CLEVR) that dealt with similar but different concept learning and reasoning challenges. What each research group considered as sufficient generalization indication was generally different; some of them were more concentrated on numerical generalization, whereas others associated it with the ability for disentanglement of features and user-defined compositionality. In 2019, a new dataset called Collision Events for Video Representation and Reasoning (CLEVRER)^60^ dealt with benchmarking videos instead of images. It is mostly concentrated on the recognition of causal relationships and is closely related to physical reasoning and action prediction benchmarks that were published around that time. Both CLEVR and CLEVRER evolved in a direction that includes questions not synthetically generated but in human language.^52^^,^^67^

One far-reaching goal is that the AI algorithm after it is trained on a benchmark dataset will contain the reasoning abilities necessary to be able to use them in a real-world dataset.^65^ Particularly in CLEVER_HYP,^64^ it is clearly stated that it is created to test the reasoning skills that a robot (following ideally natural language instructions) must possess to know the effects of its actions and by that having an important skill of human-level cognition. This has also been obvious by several AI solutions that test the learned skills mostly in computer games^47^^,^^68^.

Table 1 contains an overview of all diagnostic/benchmark datasets that are analyzed in this work sorted by date of publication. It is crucial to differentiate between benchmarking 2D images, and 3D images rendered in 2D or 3D video sequences since this affects the set of real-world tasks the AI solutions (for example, robots) will be able to solve, as mentioned previously. The size of the dataset is also characteristic, although it might be decisive for the skill comparison of the AI algorithms to manage to acquire them with the least amount of training data and human-expert priors as well as the reporting of the balance between those two.

**Table 1:** Overview of synthetic datasets for benchmarking concept learning, reasoning, and generalization, sorted by date and characterized by size and dimension (2D, 3D rendered in 2D, 3D video)

Dataset	Dimension	Size	Reference	Date
CLEVR	3D to 2D	70,000; 15,000; 15,000	Johnson et al.^50^	2016.12.20
Shapeworld	2D	customizable	Kuhnle and Copestake^51^	2017.04.14
CLEVR-Humans	3D to 2D	17,817; 7,202; 7,145	Johnson et al.^52^	2017.05.10
Sort-of-CLEVR	3D to 2D	9,800; 200	Santoro et al.^53^	2017.06.05
SCOOP	2D	variable	Bahdanau et al.^54^	2018.12.30
RAVEN	2D	672,000; 224,000; 224,000	Zhang et al.^55^	2019.03.07
CLEVR-XAI	3D to 2D	20,000	Arras et al.^56^^,^^57^	2020.03.16
Kandinsky Patterns	2D	customizable	Müller et al.^58^	2019.06.03
V-PROM	3D to 2D	different splits ∼100,000	Teney et al.^59^	2019.07.29
CLEVRER	3D	10,000; 5,000; 5,000	Yi et al.^60^	2019.10.03
CATER	3D	3,850; 1,150	Girdhar et al.^61^	2019.10.10
CLOSURE	3D to 2D	sizes as CLEVR	Bahdanau et al.^62^	2019.12.12
Bongard-LOGO	2D	9,300; 900; 1,800		2020.10.02
CURI	3D to 2D	500,000; 5,000; 20,000	Vedantam et al.^63^	2020.10.06
CLEVR_HYP	3D to 2D	5,000; 1,000; 1,000	Sampat et al.^64^	2021.04.13
Super-CLEVR	3D to 2D	20,000; 5,000; 5,000	Li et al.^65^	2022.12.01
CLEVR-X	3D to 2D	56,000; 14,000; 15,000	Salewski et al.^66^	2022.04.05
QLEVR	3D to 2D	70,000; 15,000; 15,000	Li and Søgaard^1^	2022.05.06
CLEVRER-Humans	3D	1,108 splits as CLEVRER	Mao et al.^67^	2022.10.14

The size column, when it contains three separated numbers, contains the size of the training, validation, and test set. In cases where there are only two numbers, it is the size of the training and test set. In some datasets, the user has the ability to generate an arbitrary amount of samples; the size is then “customizable.” In situations where the splits can have variable size, this is denoted with the value “variable.” In many cases, the size and splits are equal or proportional to the primary dataset.

### Design directions for concept learning and reasoning benchmark datasets

In the design phase of those benchmark datasets, the following eight directions must be carefully thought of: concept distribution, generalization, complexity, bias, ground truth conformity, ambiguity, causality, and domain transfer/extension. For the vast majority of those dimensions, there is either an explicit or implicit metric that is used to quantify the extent to which it is present in the benchmark dataset by construction. This is not to be confused with the characterization of the AI algorithms’ abilities in recognizing those features properly.

#### Concept distribution

This dimension is strongly related to the one capturing biases (see section “bias”) and is basically addressing the following questions: how many samples will contain a particular concept? Are all concepts uniformly distributed along the dataset, or are some of them more prevalent? One example of the presentation of the distribution of question types in the Super-CLEVR dataset is presented in Figure 2 and the concept distribution in Figure 5. The distribution of characteristics of objects is also something that influences indirectly the concept learning procedure; therefore, a depiction as in Figure 3 is helpful.

**Figure 1:**
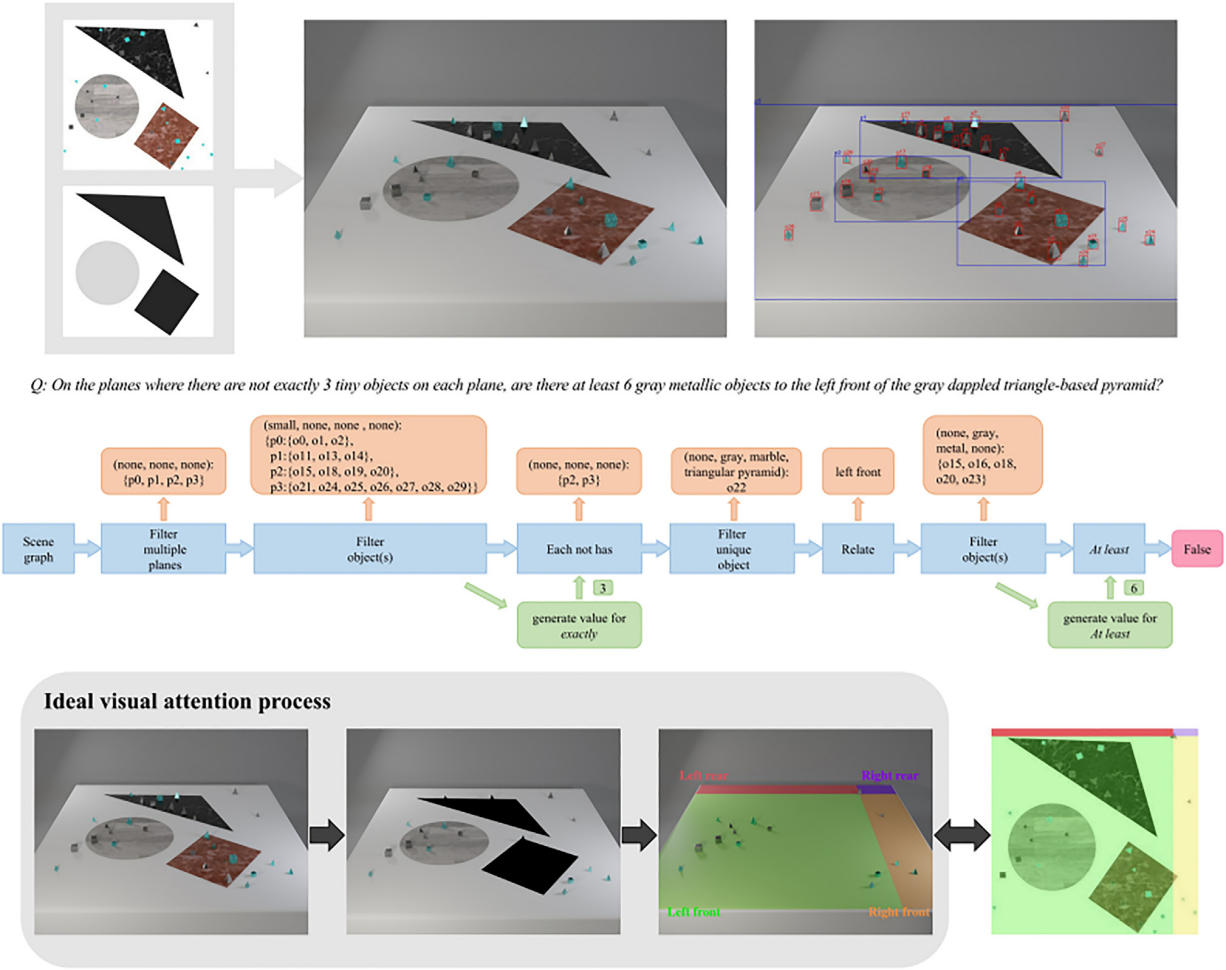
Example image and question pair from the QLEVR dataset One example image and question pair from the QLEVR dataset^1^ as well as the corresponding logical steps to answer it. The image contains objects of different shapes, sizes, colors, and textures in different backgrounds. To answer the question correctly, an AI algorithm can use the presented logical steps, although it has been shown that, in some cases, the correct answer is provided without the model following some expected reasoning sequence. For humans, such tasks are easy; for computers, they are still difficult.

**Figure 2:**
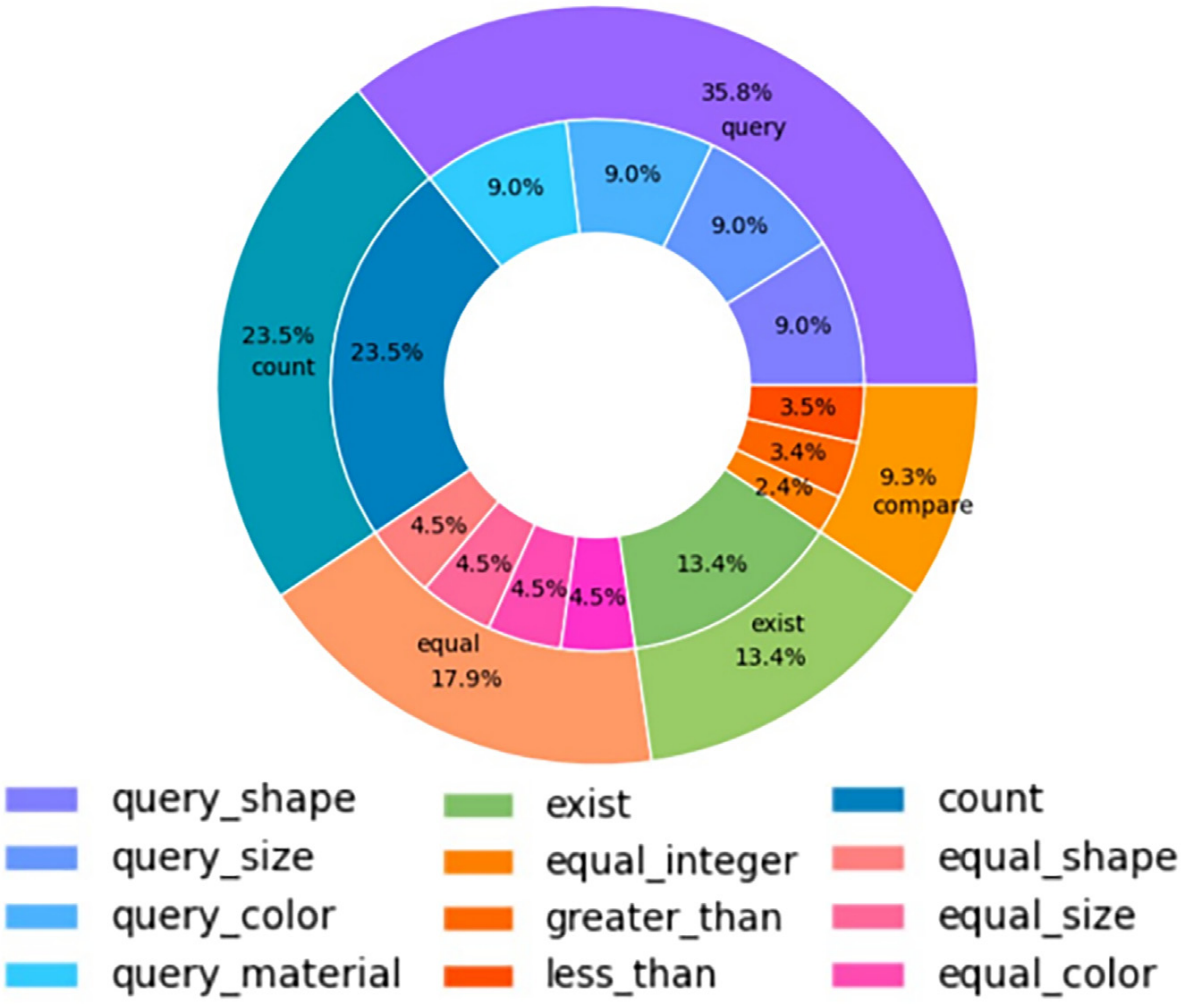
Pie chart of the distribution of question types in Super-CLEVR A pie chart of the distribution of question types in the Super-CLEVR dataset.^65^

**Figure 3:**
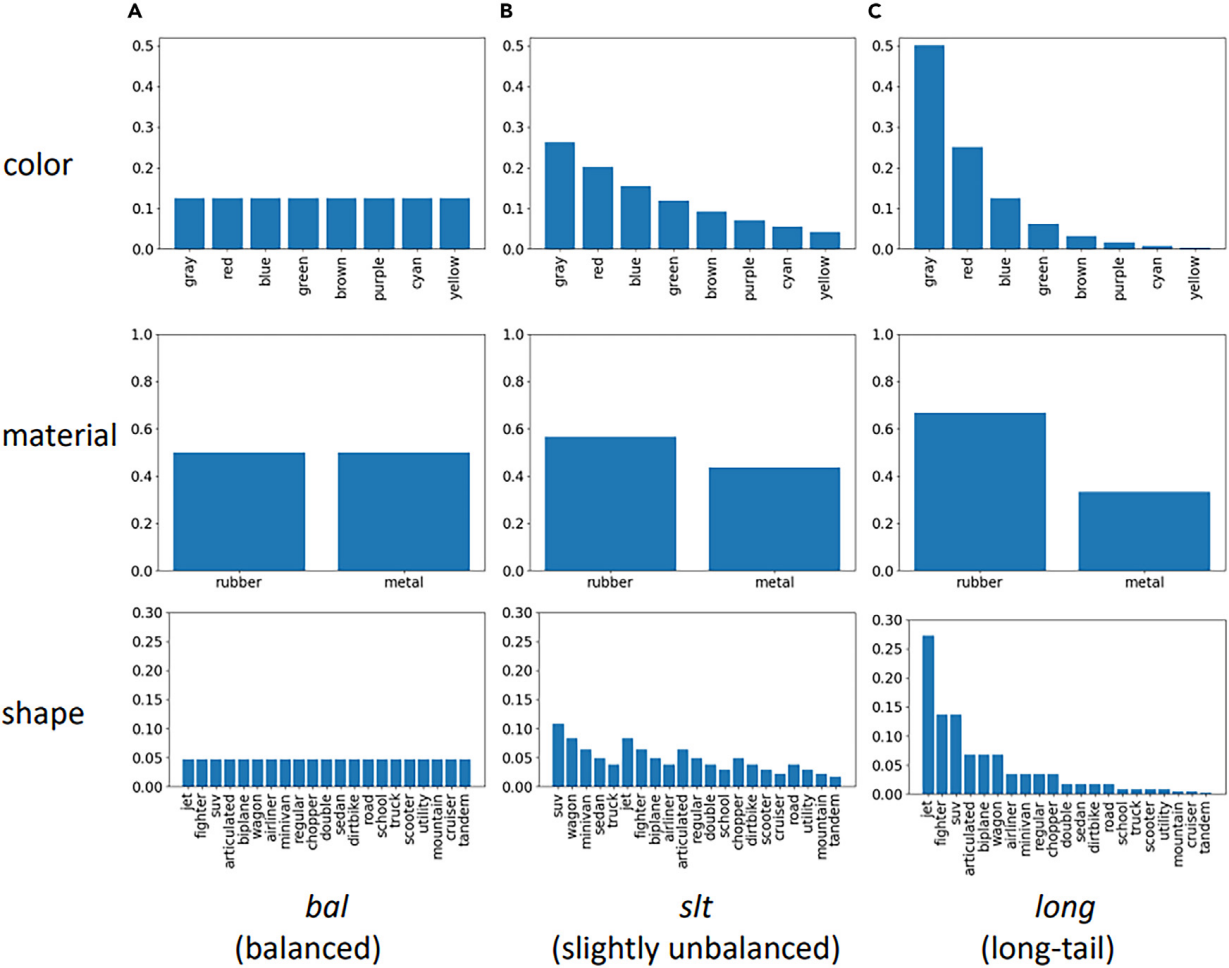
Objects in the Super-CLEVR dataset Distribution of characteristics of objects in the Super-CLEVR dataset.^65^

#### Generalization

**Figure 4:**
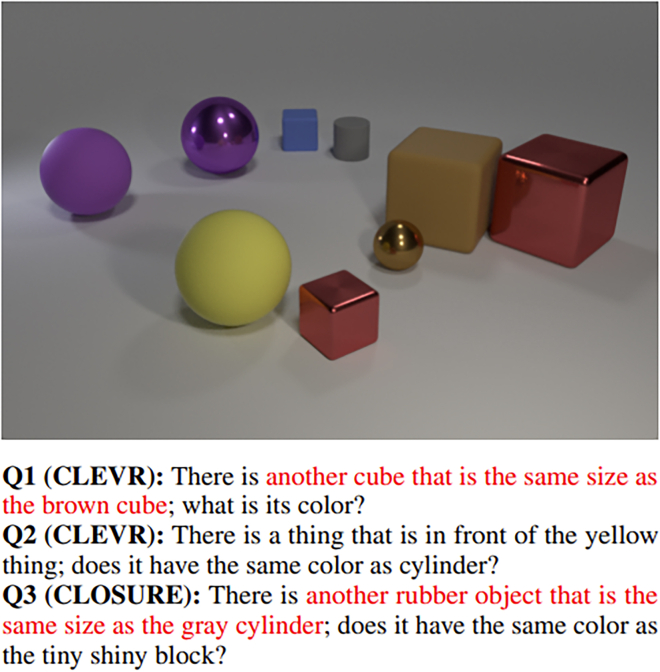
Example from the CLOSURE dataset Example from the CLOSURE dataset that extends the checks for generalization compared with CLEVR.^62^

The out-of-distribution (OOD) abilities and problems of AI solutions are topics that are mentioned in a plethora of research works. This design direction deals with uncovering the limits of unseen data constellations that are solvable. Generalization has different subdivisions that are important, such as numerical, interpolation, and extrapolation of the grasped concepts, extension to conceptually similar problems, and most importantly the degree of disentanglement as a necessary prerequisite for the emergence of novel, more complex combinations, also called compositionality. One tries to capture it by differing the distribution of data across splits—either the characteristics of image data, questions, and/or answers—and noticing if the performance will drop and, if so, by how much. The CLOSURE dataset (see Figure 4) was created under those principles to improve some deficiencies of the CLEVR dataset in this topic. The ultimate goal is the generalization to a real-world dataset that would demonstrate both domain robustness and domain shift abilities.

**Figure 5:**
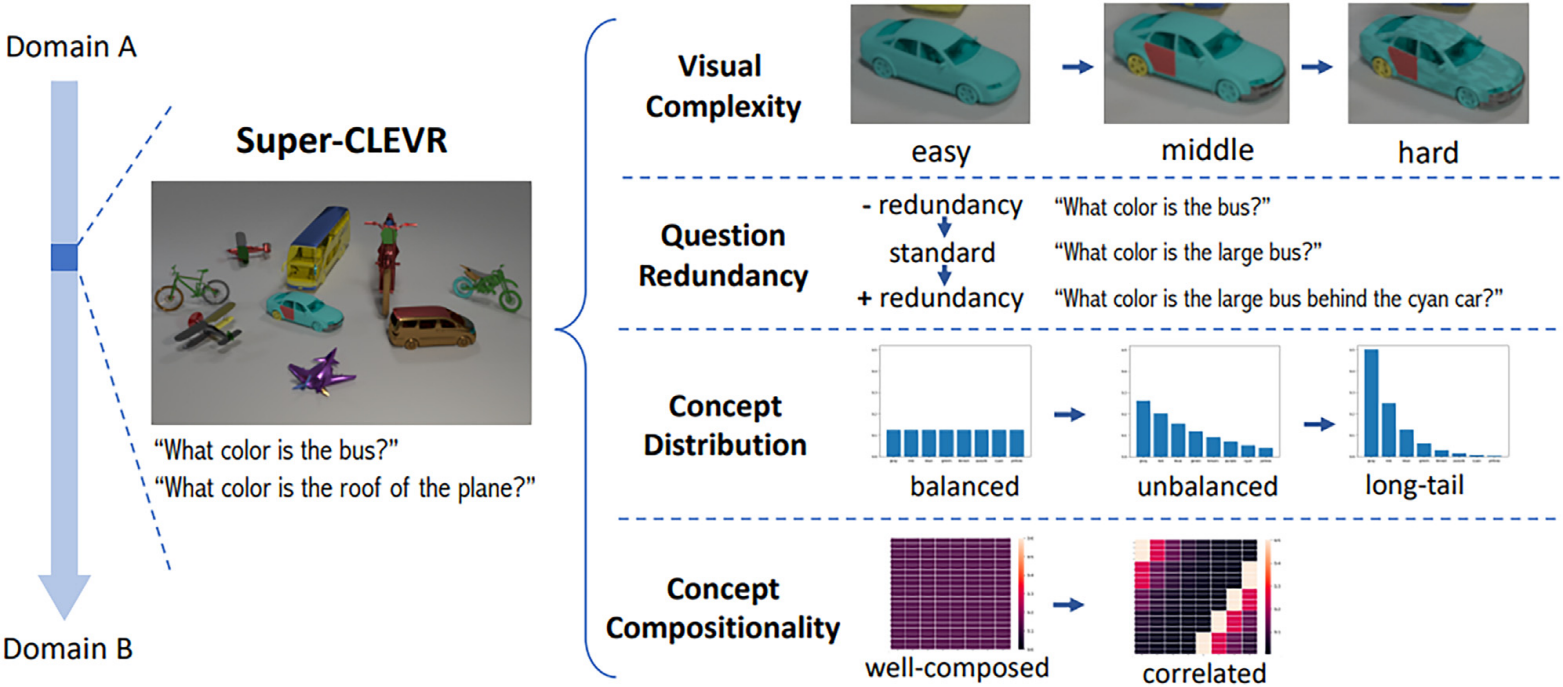
Features of the Super-CLEVR dataset The visual complexity, question redundancy, concept distribution, and concept compositionality of the Super-CLEVR dataset.^65^

The degree of generalization is typically measured by the performance of the test set or in specially designed splits; its relative drop compared with the one of the training set is the major indication of its lack.^69^^,^^70^ The synthesis abilities are put into question explicitly,^68^ and, in some cases, a heatmap of concepts co-occurrence matrix and conditional concept distribution (a concept in the context of other concepts)^65^ is presented (see Figure 5). In the Super-CLEVR dataset, the authors define the relative degrade (RD) metric as the percentage of accuracy decrease under domain shift.^65^

The design direction of compositionality is so fundamental that independent research works tackling its issues was created.^70^ It is quite clear that particularly the compositional analyses are created with a human reference in mind^71^ (and sometimes even anti-correlated with them) and that the learned model embeddings synthesis characteristics have to be expressed with a mathematical framework. The evaluation metric tree reconstruction error (TRE) is created to determine if a compositional structure is present by checking the degree of correlations between learned representations. The mutual information (MI) is between the TRE of the whole dataset and the learned representations. Newer research works deal with assessment methods that create adversarial agents, trying to stress the limits of compositional generalization with the use of zero-sum games.^71^

#### Complexity

How does the AI solution perform when increasing the number of elements that comprise the image, when there are more occlusions in the scene and the longer text of questions and answers? Tendentiously, the complexity is considered to increase when more objects, relations, and concepts are represented by the sample.^72^ The use of natural language instead of a domain-specific language (DSL) for the question-answer pairs is one of the most noteworthy complexity-increasing decisions. Correspondingly, the AI solution needs to undertake more logical steps and also more actions. An example of increasing complexity is presented in Figure 5.

#### Bias

Historically, in the earlier benchmark datasets, the researchers made a great effort to eliminate representative bias, particularly in dealing with occlusion and overlap of scene objects and events, trying to counteract them. Even since 2011,^73^ ideas about how to make datasets as rich as possible through augmentation or data gathering from the Internet showed not only that there are different types of bias but also that several counter-measures to make the data gathering as random and careful as possible fail since whole datasets have unique characteristics that can be discriminated from each other with a good performance with the use of AI algorithms. This might have led to the phenomenon that is observed in later datasets; since real-world data contain biases, an ideal situation with an unbiased dataset is considered to be unrealistic. Therefore, some biases are meanwhile per design quite “tolerable,” and only the ones that are important for real-world generalization and application (such as the rotation and scale invariances) are kept; see also the section “generalization.”

Visualization of distributions and the results of balancing processes are discussed elsewhere^74^^,^^65^ (balanced, unbalanced, long tail), and pie charts for the distribution of question types are also available,^65^ along with details with pie charts for question length and frequency distribution of quantifiers distribution.^1^

#### Ground truth conformity

All benchmarks are built to have a well-defined ground truth by construction. In most cases, the program that created the synthetic data is used as a reference for the logical steps and actions that need to be followed to reach a solution, but researchers discovered that, in some cases, the AI algorithm learned the necessary concepts and relations differently. The extent to which this can be an acceptable solution (and not the result of a spurious correlation) is something that needs to be pre-specified and co-decided under the guidance of explainable AI (xAI) methods.

The metric that can be used for this characteristic is the grounding scores for all models using attention as in Hudson et al.,^74^ or implicitly through xAI methods.^56^ The main idea in both research works is to check whether the model attends the parts of the image that are important for the concept, or that an adequate xAI method discovers that they have high (positive) relevance.

#### Ambiguity

A design direction closely related to ground truth conformity (see section “ground truth conformity”) is will each data sample belong to only one concept or can it be generated by many, each of them with a particular probability? To find a mapping between each element in a data sample and its corresponding symbolic description is commonly called grounding; ambiguity acceptance and grounding alternatives are also seamlessly increased the less detailed the specification of the questioned elements has.

**Figure 6:**
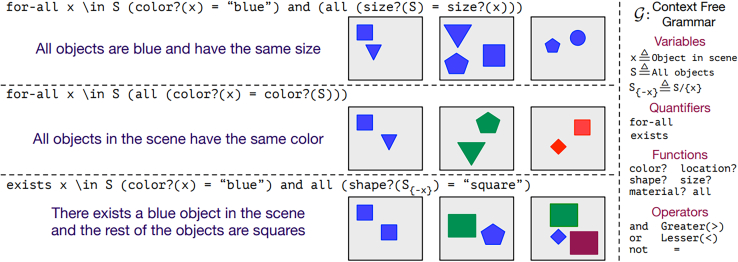
Some example images from the CURI dataset The grammar rules that created those examples are presented along with the images.^63^ Nonetheless, these rules are not unique, and the probabilistic context-free grammar can create the same images from different rules.

One of the most prevalent examples of datasets containing built-in ambiguity is concept learning under uncertainty (CURI), as seen in Figure 6. Each sample of this dataset could have been generated by several different concepts with some pre-defined probability.

#### Causality

The benchmark datasets are tendentially evolving from static scene images with the corresponding descriptions and/or question-answers to sequences of images (i.e., videos). The understanding of change detection, long-term effects, and the exhibition of spurious correlations over a sequence of samples is vital for temporal and physical reasoning, which in turn is a conceptual predecessor for causality benchmarking.

**Figure 7:**
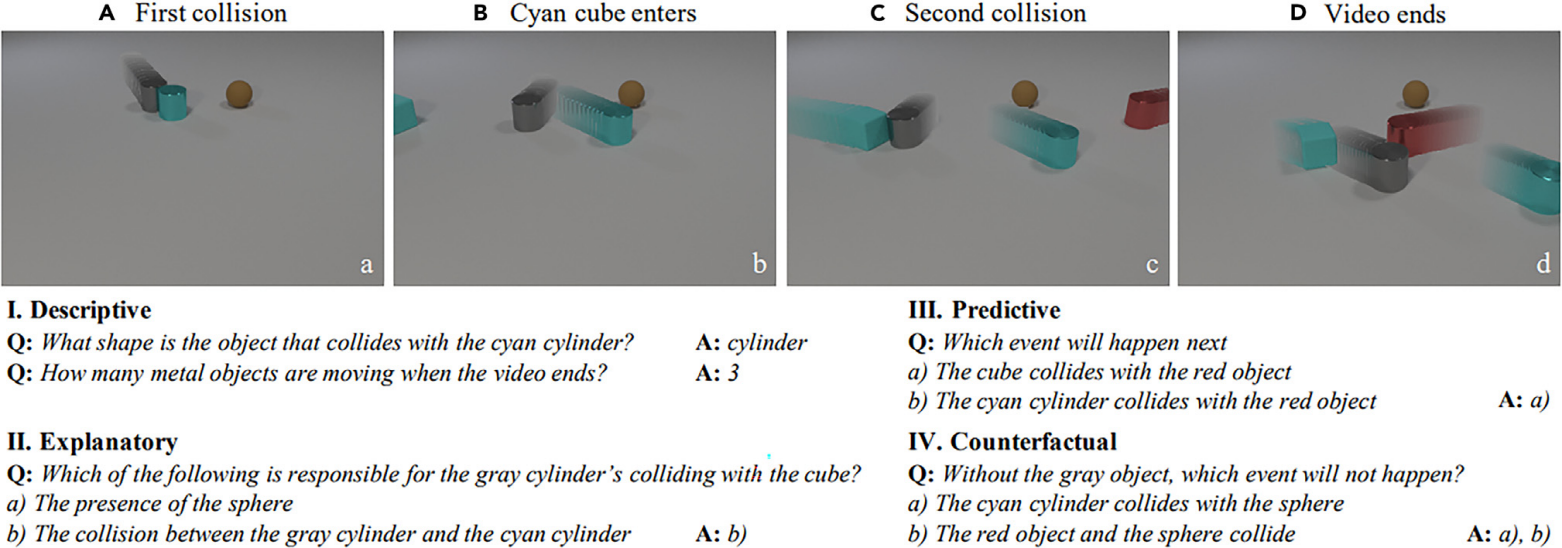
Four frames of a video containing two collisions in the CLEVRER datasets The corresponding descriptive, predictive, explanatory, and counterfactual questions are shown.^60^

Figure 7 contains a representative example of four frames containing two collisions, along with the corresponding questions and answers. The metric for measuring the acquisition of causal abilities is on the one hand the performance on the test set, but, especially in this case, the answering of dedicated counterfactual questions (along with descriptive, predictive, and explanatory ones). Answering “what-if” questions and “imagining” counterfactual scenarios are the basic indicators for causal reasoning to some degree.

#### Domain transfer/extension

**Figure 8:**
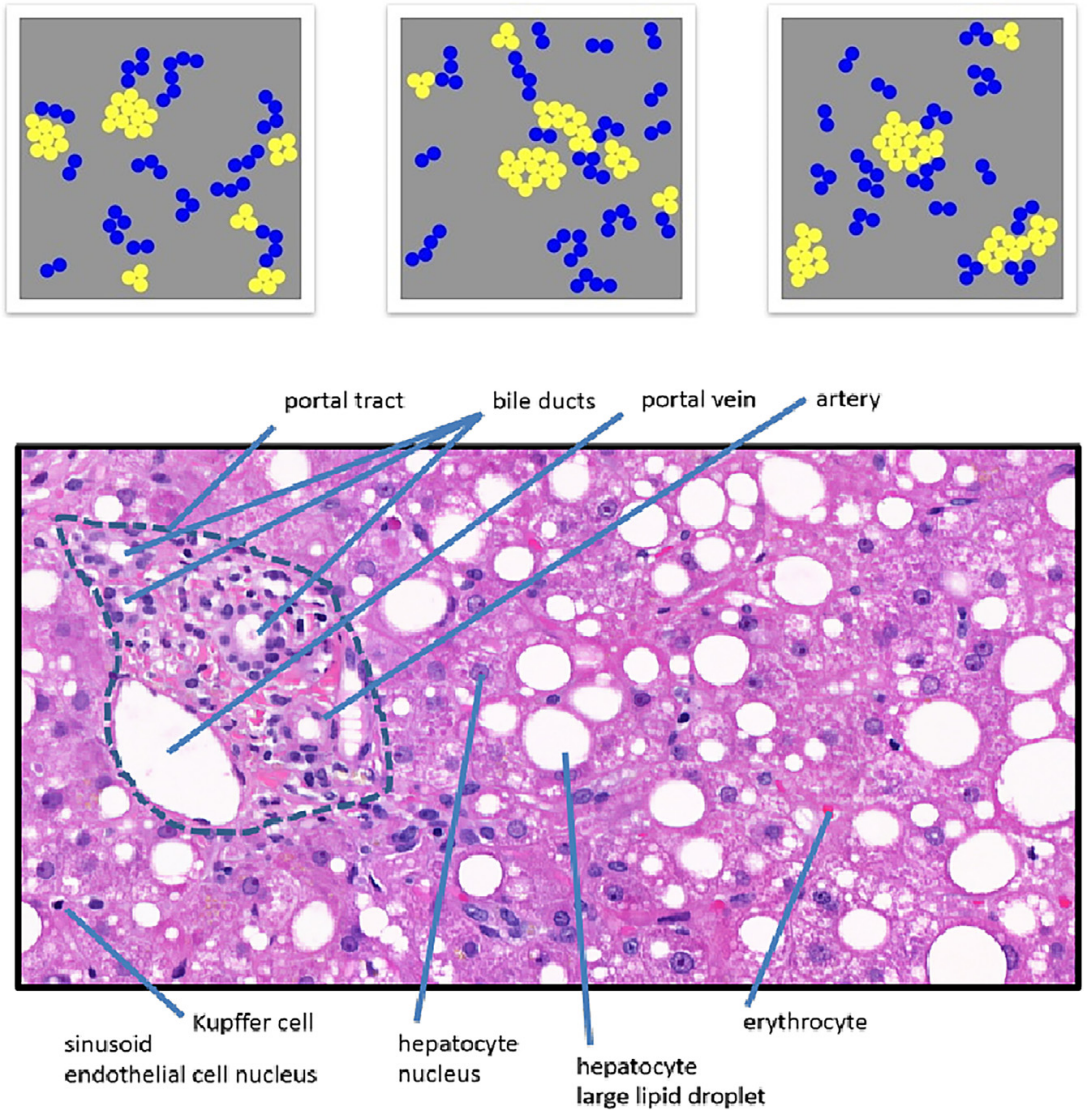
Kandinsky Patterns Inspired from the work of pathologists^75^^,^^76^ and named after the famous painter Wassily Kandinsky.^58^

Each of the datasets is inspired by a real-world domain that has a concept learning and relation uncovering problem that is unsolvable by current state-of-the-art AI models. Some of them contain conceptual challenges that should generalize for game-playing, medicine, and robot applications. Others come from the perspective of evaluating the logical and cognitive abilities of AI algorithms compared with those of humans. One representative dataset is the Kandinsky Patterns, which was created with the inspiration and the goal of transfer in the medical domain, as seen in Figure 8.

**Figure 9:**
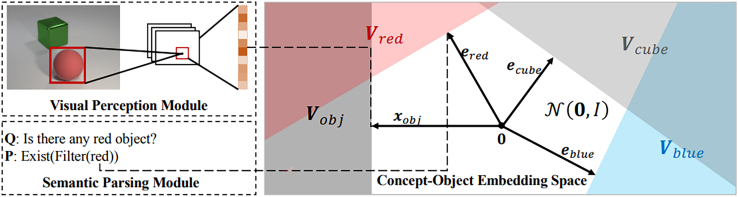
Concept and object vectors Concept and object vectors embedding space of concept objects; the model learned the embedding of an unseen concept in Han et al.^77^

In other works that use the CLEVR dataset, the extension to a newly created DSL, or even natural language,^47^ is an indication of acceptable domain transfer as long as the performance of the model in the new language is acceptable. The extension to images of the Minecraft game^68^ or Lego game constellations are also evidence for the applicability of the solution to similar visual domains. Furthermore, meta-concept learning of a completely new concept in the embedding space of concept-objects^77^ is shown in Figure 9.

### CLEVR

CLEVR^50^ is one of the most used diagnostic/benchmark datasets that were used for visual question-answering systems and was also challenged and improved by diverse research groups.^78^ The scene in all images of the dataset contains preliminary simple three-dimensional geometrical objects (cubes, cylinders, spheres) of various colors, sizes, and textures, each time in a different constellation. For each image, there is a set of possible questions that were synthetically generated with a functional program; each of them is created by a chain or tree of reasoning functions encompassing mainly relations, logic, existence, uniqueness, counting, and comparison concepts. By that means, the ground truth is available on demand and can be used to verify the performance of neural networks that learn the corresponding concepts, which are presupposed for resolving the reasoning tasks and correct answering of questions. Surprisingly, there were technical solutions such as NMNs (described in section “NMNs”) that provided the expected answer, without necessarily following the ground-truth-defined reasoning sequence.

The generalization aspect was considered thoroughly in CLEVR; rejection sampling was incorporated to make sure that the answer distribution is uniform. Furthermore, the question’s textual content can impose biases, since long and complex questions contain more concepts and will need longer reasoning paths by trend. The neural network architectures of that time relied on image and textual alignment of embeddings and therefore showed poor generalization results. Even in cases where the test image was only differing by one attribute value from an image that was encountered in the training set, the performance was not satisfactory. This indicated the fact that deep-learning models do not learn and contain disentangled representations of attributes and objects, which explained the drop in performance of those models on unseen images and questions that were composed by the same distributions as the ones in the training set.

#### CLEVR’s successors

An expansion of CLEVR called CLOSURE was invented in 2020 by Bahdanau et al.,^62^ together with a proposed architecture that is inspired by NMNs,^50^ analyzed in section “NMNs),” as well as symbolic approaches, described in section “neuro-symbolic methods.” They observed that CLEVR does not have uniformity in the label of the training and test set, comparison questions only use spatial referring expressions, and other related artifacts that made the path for the creation of an enhanced synthetic dataset. The researchers argue that the questions in the test set must have a different distribution—even if the image distribution is the same—and it should contain new combinations of already-learned semantic and syntactic components.

Another variation of the CLEVR dataset concentrating on relational reasoning is Sort-of-CLEVR.^53^ This is a distilled 2D version of CLEVR containing images that always have a fixed number of objects, the only attributes are colors, and the questions have fixed lengths and consist of relational and non-relational questions.

Another variant, called CLEVR-Humans,^52^ contains the same images but uses questions that are posed by humans, have much more diversity, and are linguistically more complex than the synthetically generated ones.

Newer benchmark datasets are created with the goal of exercising machine learning models on CURI.^63^ Concepts can be ambiguous and are no longer rigid labels; for example, one image can belong to many different concepts. Each concept is expressed by a probabilistic context-free grammar containing (among others) logical operators and comparisons. The number of samples in the dataset is pre-defined so the acquisition of a concept has to be made with limited data. Furthermore, new challenges arise from the comparison of different data modalities, such as images, sounds, and symbolic schemes in textual form. The comparison of the different performances, representations, and appropriate models sheds light on the commonalities and differences between the input data and the task itself.

The data are according to pre-defined hypotheses and either satisfy a concept (positives) or not (negatives) with a particular probability. Current representation learning methods^79^ that are used to embed the states of reinforcement learning (RL) environments are also based on this scheme of positive and negative examples created by corruption of the positives. The compositionality gap is measured by the comparison between an ideal Bayesian learner that has access to all the hypotheses. Furthermore, stronger and weaker generalization tasks are defined by the targeted choice of negatives that have partial overlaps with the concept of the positives. Generalization is tested by strategically designed splits between the data that are used for training and the ones that comprise the test set. For example, to evaluate how well the generalization is accomplished, easier concepts (with smaller prefix sequence length) are present only in the training set, whereas the test set contains only complex concepts.

**Figure 10:**
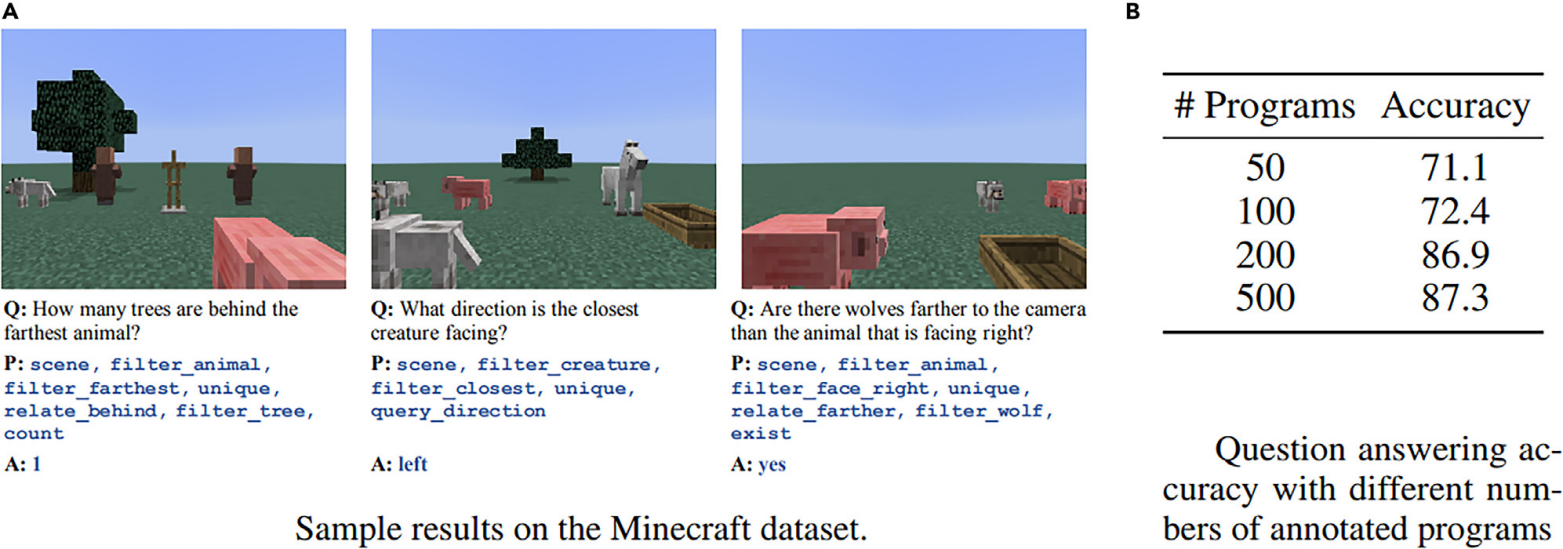
Accuracy of the NSVQA system Accuracy of the NSVQA system on Minecraft images.^68^

Super-CLEVR^65^ is a benchmark dataset extending CLEVR that specializes in exercising AI solutions with respect to (w.r.t.) domain robustness and generalization. Instead of focusing solely on the correct creation of balanced dataset splits or counterfactual samples generation of other datasets,^80^ this dataset is focused on first dividing domain shift into four components, the first being visual complexity by extending CLEVR’s 3D objects with more textures and parts. Second, questions are created with more redundancy in the provided information as humans do, and that increases the number of correctly identified logical steps and the probability of error in the AI solution. The third component targets the concept distribution as far as the number of samples that contain (or do not) a particular attribute; balancing those is dealt with by varying the distribution from uniform to long tailed and measuring concept distribution shifts. The fourth aspect is concept compositionality, which deals with the ability of an AI solution to disentangle attributes and objects from each other and being able to correctly identify a combination thereof that does not occur as in the training set. For each of the four components, the dataset contains three parts characterized by the controlled and increasing complexity of those. The differences in model prediction performance (also called RD) are the indicators of domain robustness; this was effectively made with the implementation of a Probabilistic Neural-Symbolic VQA (P-NSVQA) algorithm (see section “neurosymbolic methods”; Figure 10), which performed better than other baselines and older neuro-symbolic models.

Another benchmark dataset, namely QLEVR^1^ (Figure 1), that extends CLEVR concentrates on increasing the complexity of quantifiers and contains questions composed of several combinations thereof. This is made in an attempt to approach human natural language questions even better and prerequisites even more complex image constellations than CLEVR. The synthetically generated images can contain in this case more than one background with different textures, and the objects lie in groupings. The inventors have created dedicated checks to ensure that questions are not ill-posed or trivial; this is even more necessary in this dataset where the complexity is much higher and therefore the questions are more prone to being always true or “arithmetically impossible” in the sense that no reasonable grounding is possible. Furthermore, to ensure that the answer distribution is balanced, there were much more questions than images. The AI solutions that were used include text-only models as base cases and further checks to detect spurious correlations in the question’s text and also to uncover the necessity of the image information for the correct answering of the posed question. As also observed in other benchmark datasets, the more detailed the specification of the questioned elements, the more prone the AI solutions are to an erroneous answer; whenever an exact match is required, the problem is more difficult than the recognition of, e.g., “larger than,” “lower than,” “all,” or “some” solution sets. It is apparent that, where less reasoning is required, as, e.g., in cases where less attribute or relation recognition is necessary, the performance is generally higher. The most effective AI solution includes the memory, attention, and composition (MAC),^69^ which is described in section “AI solutions.”

CATER^61^ is one of the first benchmark datasets that deals with video and spatiotemporal understanding accordingly. It is referred to by the inventors themselves as a diagnostic/benchmark tool, and they suggest that it can be used for other tasks apart from benchmarking long-term reasoning. The models are exercised in their abilities in solving four classes of tasks, the first being primitive action recognition without the explicit use of an operator or hand (not meant for action recognition of an imaginary robot arm). The second task is more difficult and deals with the correct recognition of compositional actions; this deals with pairs of actions whose duration interval overlaps at a particular time point or interval. Recognition of completely hidden or partially occluded objects is also an interesting problem and it inspired the last two tasks, one dealing with adversarial target tracking and the last one with the containment of one dedicated object called “snitch.” The performance of the AI models used on this benchmark was not in accordance with the one they had in real video sequences most of the time; nonetheless, all of them struggle with the effective discovery of hidden elements, particularly if their movement was active until the last video frames since they did not have the opportunity for long-term inference. Models that used just aggregation functionality could not effectively process temporal information, whereas ones that contained LSTM^81^ components were more capable of reason about it.

Although one of the stated goals is the discovery of causal relation, the researchers do not see this dataset as comparable with or in the same category as CLEVRER,^60^ described in section “CLEVRER,” but as an extension of CLEVR for frame-by-frame classification dedicated to action recognition. In some sense, it has similarities with CLEVR_HYP and CLEVRER, which is described below, since action recognition and understanding the effects thereof are issues also tackled by those benchmark datasets. As far as the elimination of biases is concerned, the inventors have the opinion that, since videos of the real visual world contain them, the benchmark datasets should reflect that. Benchmarking is there to shed light on what models misunderstand by creating a challenging dataset where occlusion, duration of events and their overlap, and degree of camera motion are parameterized so that humans can understand those limitations.

CLEVR_HYP^64^ is a benchmark dataset that, although not designed to exercise reasoning skills for video sequences, can be considered a forefront to CLEVRER,^60^ described in section “CLEVRER,” in the sense that it deals with actions that change the image’s scene. The dataset’s image’s objects do not have substantially different characteristics compared with CLEVR, apart from five pre-defined spatial relations. The main difference lies in the questions; for an AI solution to be able to answer them correctly, it has to imagine the correct sequence of actions by sequencing events that happen before and after the current image’s scene. There are four types of actions that are considered executable by a robot and can influence either one particular object or the whole scene. Some sort of counterfactual “what-if” hypothetical scenario conceptualization is necessary as well as “physical intelligence” that one also encounters in the solutions of Bakhtin et al.^82^ without it yet being a benchmark that tests causal sequence events as CLEVRER^60^ (see section “CLEVRER”). Nevertheless, all scenes until the last one that manifests the correct question’s answer have to be physically plausible. The posed questions are more complex than the ones in CLEVR by the use of synonyms and paraphrasing, which also contributed to the prevention of over-fitting. As in CLEVR, the longer the question, the higher the number of actions that need to mentally be performed to answer the question properly. The researchers took care of dataset balance by making all answer choices uniform and cleaning up ill-posed or “degenerate” questions that do not have any corresponding grounded objects or contain ambiguity in this manner. The NS-VQA^68^ described in section “neuro-symbolic methods”was used here among other baselines and provided acceptably good performance.

CLEVR-X^66^ is a dataset that provides explanations for a visual question-answering task. The novelty of CLEVR-X is that it extends the CLEVR dataset with natural language explanations. The authors state that natural language explanations allow for a better understanding of the reasoning process and thus enhance the transparency of the system. Further, the CLEVR-X explanations do not simply describe all image elements but only those relevant to the input question. This is done by first tracing the functional program, relevance filtering, and finally generating the explanation. CLEVR-X explains all relevant elements, thus all objects that are needed to answer the question are filtered and explained. To keep the explanations as short as possible, only the object properties asked for are deemed relevant, and thus are included in the explanations. Explanation templates with placeholders are used for the explanation generation. Since they should be kept short and simple, repetitive expressions are aggregated using numerals. Thus, multiple different explanations are generated by using different templates, random sampling of synonyms, and object order randomization. A subsequent user study showed that the explanations of CLEVR-X are complete and they match the questions and images of the CLEVR dataset. It was shown that the generated explanations for easier question-and-answer categories exhibit a higher quality than for other categories, whereas the explanations for counting problems showed the worst performance.

CLEVR-XAI^56^^,^^57^ is a benchmark dataset for the ground-truth evaluation of neural network explanations, based on CLEVR.^50^ CLEVR-XAI proposes methods to generate a visual ground truth, based on the CLEVR generator, as well as quantitative metrics for evaluating explanations. The CLEVR-XAI evaluation set is split into two types, namely simple and complex questions. Simple questions (CLEVR-XAI-simple) focus only on one single target object and pose queries about the object’s attributes without any inter-object relations. Complex questions (CLEVR-XAI-complex) on the other hand contain all question types that can also be found in the original CLEVR dataset. Thus, multiple objects within the image can be relevant to a question. Ground truths are generated for both of these types. Since the simple questions do only focus on one single target, two ground-truth masks are generated: one that only identifies the target and another that identifies all objects. These masks are created by setting the corresponding object’s pixels to true and the remaining pixels to false. The complex questions make use of four individual ground-truth masks. One mask identifies a unique object, related to the posted question. The ground-truth unique first-non-empty mask returns all unique objects from the first-non-empty set, while the functional program is iterated in reversed order. The ground-truth union mask is a superset of unique first-non-empty and is still related to the question. It includes a union of all sets of objects returned by every function of the program. Similar to the simple questions, there is one ground-truth mask that contains all objects of the scene. Most xAI methods generate a heatmap with three channels, which is then pooled by the authors into a single-channel heatmap. This resulting heatmap is then used to evaluate the accuracy of an explanation by assuming the most important parts of the relevance lie within the ground-truth mask.

### CLEVRER

CLEVRER^60^ is a dataset that extends CLEVR (described in section “CLEVR”) with the goal of exercising temporal and causal reasoning abilities of neural networks. The dataset is not composed of static images but of videos that depict collisions between the pre-defined objects, which, as in CLEVR, have a pre-defined set of attributes and appear in different constellations. To answer the questions provided in the dataset correctly, the neural networks have to be able to reason counterfactually, in terms of “what-if” and “what-if not” a collision event would happen (or not) and explain which dependencies between positions and occurring events exist. Recognizing the motion of the moving objects in the videos helps the models maintain non-static but constant information about the involved objects. Causal relation recognition requires separate object representations, whereas causal reasoning could be overtaken by symbolic logic (see section “neuro-symbolic methods”), resembling roughly the system 1 and system 2 division of cognitive abilities.^83^^,^^84^ As in CLEVR, counteracting bias was supported by ensuring that each possible answer to each question is valid in the same number of images.

As for the purpose of CLEVRER, the ultimate goal is to evolve toward real data scenarios that will be of more practical use to the corresponding scientific communities and industry. It has substantial similarities with physical reasoning benchmarks such as Physical Reasoning (PHYRE)^82^ and CLEVR-Change for change detection^85^; nonetheless, those benchmarks concentrate substantially on the understanding of physical laws and not causality specifically.

#### CLEVRER’s successors

The CLEVRER-Humans^67^ is a human-annotated dataset of physical event descriptions and their causal relations. It extends the CLEVRER dataset by incorporating human knowledge and the way humans tend to describe physical events and the corresponding causes. Instead of basing the causal relationships on conservation laws, heuristics and counterfactuals, human annotation, and judgement about potential causal dependencies are collected through a user study, in the form of a question-answer survey. Each participant indicates whether the events, as stated by the CLEVRER dataset, are correct and, if so, indicates the level of causation (1–5) between those events. The dataset introduces the challenge of NLU, combined physical scene understanding, and causal reasoning. The goal of CLEVRER-Humans is to show the difficulty of human reasoning due to different linguistic usage and graded judgements of causation. It accomplishes that by taking into account natural language descriptions such as “because” or “responsible for” and using them for the physical grounding of chains of events. The internal generation of a causal event graph (CEG) helps the dataset generation system to the model human annotation of events and their degree of causation, while at the same time being time saving because this only has to be constructed for a limited number of cases; the rest can use a neural network that outputs human-like annotations.

The CLEVRER-Humans benchmark dataset achieves two design goals: first is the diversity between physical event descriptions (nodes of the CEG) with natural language (NL) and, second, density over the edge labels, which are also annotated by different users. Like other datasets, there are also built-in sanity checks of the annotations for grammar consistency, referred to object(s) existence (viable grounding) and re-balancing of verb usage since verbs are indicative of events. Particularly for re-balancing, human users are adamant in their contribution of ensuring uniformity since most of the events involving two objects do not have a causal relationship between them. Separate gated recurrent unit (GRU) networks with attention^86^ used single- and pairwise-event description generation where the input is the trajectory of one object or both in cases of approaching, collision, and moving together in a pair correspondingly. The challenges posed by the high diversity of this dataset w.r.t. CLEVRER’s vocabulary size or the limited training set size that leads eventually to over-fitting are not overcome by state-of-the-art neuro-symbolic AI solutions, even if they are trained or pre-trained on the CLEVRER dataset.

### Other diagnostic/benchmark datasets

KANDINSKY Patterns^58^ are named after the Russian painter Wassily Kandinsky and their building blocks are three simple shapes (circle, triangle, square), which can vary in color (blue, red, yellow), size, and position, similar to an abstract painting by Kandinsky.^31^ With these basic visual elements and optional restrictions, e.g., Kandinsky Figures contain exactly four geometric objects, a set of all possible Kandinsky Figures is defined, which is divided into two subsets, by either a mathematical or a NL statement; e.g., “All elements in a Kandinsky Figure are red.” The two subsets, one for the true and one for the false statement, are then called Kandinsky Patterns. A Kandinsky Pattern thus represents a specific concept, which can range from very simple ones, e.g., the color red, to relationships between quantities, positions in space, and Gestalt principles.

Tasks to be challenged by a specific Kandinsky Pattern are how to explain a Kandinsky Pattern, if only a limited number of Kandinsky Figures are known, and how to generate an NL statement, which is easily understandable and equivalent to the machine explanation (classification algorithm). Furthermore, Kandinsky Patterns can be used to investigate the generation and refinement of a hypothesis when a series of false and true Kandinsky Figures are received alternately. Since Kandinsky Patterns can be described both in a mathematical formalism and by humans, Kandinsky Patterns lend themselves to comparing the process of learning (hypothesis refinement) between humans and machines^80^^,^^87^^,^^88^

The SAR Altimetry Coastal & Open Ocean Performance (SCOOP) dataset^54^ is one that is not tailored particularly to concept learning but concentrates on exercising the generalization capabilities of NMNs^45^ (see section “NMNs”) with different layouts that do not need prior knowledge about the task. The proposed dataset contains images with letters and digits, and the reasoning tasks encompass only spatial relations. The experiments showed that the architecture of NMNs played a substantial role in their generalization capabilities; modules that are organized and connected in a tree structure have an excellent generalization performance, comparable with models that use prior knowledge, whereas a set of modules structured as a chain fail just because of that reason. It is a key insight that, to achieve compositionality, apart from parameterization there has to be successful specialization between the modules and the layout must be inducted adequately.

The Raven progressive matrices (RPM) test^89^^,^^90^ is a non-verbal test, invented by psychologists mainly to test the recognition of relations between objects and attributes. Each test is composed of a 3×3 matrix; each row of this matrix contains images having the same relationship with each other. The relations consist of logical operators, comparisons, and counting objects, which is still a challenging task for current state-of-the-art neural networks. The questioned entity must choose the adequate answer from a set of candidate images; this constitutes a major difference from the rest of the datasets and cognitive tasks explained in this section. The images consist of simple abstract shapes, selected from a closed set.

A smaller but more diverse dataset following the main principles of RPM, called Relational and Analogical Visual Reasoning (RAVEN),^55^ was used to exercise the reasoning capabilities of relation networks (see section “relation networks”). The creation of each image is made with the use of more structures and instantiations, as well as following more diverse types of rules, although they are hierarchical. Furthermore, researchers did not only compare the performance of the models w.r.t. ground truth but also with human performance.

A new benchmark for visual reasoning for real images that goes beyond testing the generalization capabilities of non-abstract scenes is visual progressive matrices (V-PROMs).^59^ Although the style of the posed problem follows the structure of the RPMs, meaning that the input is still a 3×3 matrix of images connected by a particular relation at each row, the set of possibilities is open and the data are sampled from the Visual Genome (https://visualgenome.org/). Teney et al.^59^ created a detailed list of data splits with interpolation/extrapolation, held-out objects, attributes, and relationships, since the neural networks that they used struggled with OOD data. An important requirement for the machine learning solutions that will solve those tasks efficiently is also the computation of a simple abstract description that will be used as a generative explanation for the discovery of the correct image.

The Bongard-LOGO benchmark dataset^91^ has as starting point the Bongard problems (BPs).^92^^,^^93^ The Bongard problems are composed of a set of visual concept learning tasks, each of them defined by two sets of image examples that need to be differentiated. The first one is called positive and the second one is negative. All images of the second set do not obey the concept of the first. A textual description of the concept that generated the image sets is desirable; nevertheless, the authors state that there are concepts that are not easy to be expressed even by humans. Therefore, it is not a central point of this dataset, since a human or an algorithm could just state if an unseen image belongs to the same concept that generated a fixed number of others, even without explicitly stating what concept that is.

The two sets of image examples have a small number of images created by programs written in the action-oriented LOGO language.^94^ There are fundamental differences between these datasets and CLEVR, CLEVRER, and CURI. First, although the generated images are composed of basic elements, such as strokes, have different attributes, and belong to different categories, they should not be differentiated by them. There is only one object in the image, but the perception must be rotation invariant and scale invariant. Second, the fact that different images can be generated by different concepts is not perceived as ambiguity as in CURI but as a contextual hierarchical difference. The context is defined by the rest of the positive and negative images in the dataset, and one of the main hypotheses is that current pattern-recognition machine learning models have a context-free implementation policy.

There is one notable similarity between the Bongard-LOGO problems and the third challenge of the Kandinsky Patterns. A set of (typically smaller) objects are not perceived individually but as a whole, provided that they have a recognizable arrangement. In this case, there is a trade-off of concepts that are made by analogy making; the image description is not made by the listing of those objects but by the description of the form that is created by all of them.

A 2D dataset that has several similarities with the Kandinsky Patterns is ShapeWorld.^51^ The images do contain abstract shapes and the captions are synthetically generated by following the rules of a pre-defined grammar. This grammar defines a one-to-one mapping of entities to nouns, attributes to adjectives, and relations. The researchers did not proceed to evaluate the performance of state-of-the-art neural networks and comparison with human performance. Furthermore, the dataset bAbi for text comprehension^95^ also follows several principles like the aforementioned ones but targets only textual data. Parallel research for the generation of synthetic datasets for physical^82^ and mathematical reasoning^96^ is currently evolving.

### Requirements and characteristics of the synthetic datasets for benchmarking concept learning, reasoning, and generalization

All aforementioned datasets have some commonalities that emerge from the principles that help the quantification of reasoning abilities and extension of skills. The generalization power of visual question-answering and concept learning systems is the main characteristic that needs to be quantified by well-designed splits of the dataset.^44^ Adding noise as well as variability in the images is a first step toward improving cross-dataset generalization, even if the learned models do not have increased performance^73^^,^^97^. Appearance variability is ensured by gathering data from independent resources,^35^^,^^38^ and captions must be reviewed by several people^35^^,^^36^.

Recent research by Keysers et al.^71^ provides directives on how to systematically construct splits of synthetic datasets that have a primary goal to measure compositional generalization and not some domain adaptation. According to their research, each different training-test split consists of a different compositionality experiment; they exercised different encoder-decoder architectures and showed that cases with overall very high accuracy have a significant drop in performance (reaching even values as low as 20% accuracy) for carefully designed splits. They adapted an already-created textual dataset composed of very few atoms and rules, thereby enforcing that complexity will only emerge as a result of rule composition. Desired properties of benchmark datasets should be that atoms and their distribution are similar in both training and test sets, but the distribution of the compositions is at the same time as different as possible (as measured by the Chernoff coefficient^98^). The designed dataset, as well as the splits, do exercise generalization abilities of previously invented ones, such as extrapolation and number of patterns. Nevertheless, the images in each split are not generated by fulfilling only one criterion but several at the same time.

The researchers did experiments with current state-of-the-art machine learning models that tackle few-shot problems, and use meta-learning principles and symbolic methods to solve the aforementioned tasks. Furthermore, they conducted studies with humans of two levels of expertise to compare the performance of classification. Thereby, they showed that even the most progressed machine learning solutions did not perform as well as humans; this is an indication of the lack of concept learning capabilities compared with humans. For this type of research, it is important not to find a solution to a particular subset of problems—since some Bongard problems are already solved—but an effective solution that will encompass all posed problems.

## AI solutions

### Requirements and characteristics of the AI solutions that effectively solve the benchmark datasets

All AI algorithms that will be presented in this section have to fulfill some requirements to be successful in achieving the reasoning capabilities necessary to have acceptable performance on the aforementioned benchmark datasets. Ideally, the invented model architectures learn disentangled representations of the concepts at training time and then they are able to compose those concepts at test time, even if those are extremely rare or even physically impossible in real-world settings. Furthermore, they should be able to interpolate, extrapolate, and obtain some abilities of zero-shot learning (for example, being able to count more objects than the ones encountered in all images of the training set or to recognize a never seen before color). A neural network is considered to be even more capable if the training set contains only a few of the possible combinations.^54^

This section lists and describes the main directions of research that are used currently in concept and representation learning. The list is by no means exhaustive but it is represented as far as categorization is concerned; variations and improvements of (what is considered to be) the central idea, architecture, and overall solution strategy can be. Although distinct, they are sometimes entwined since, in several cases, components from one technical solution are used in another, usually with an adequate adaptation and improvement.

### NMNs

NMNs^45^^,^^99^^,^^100^ were the first attempt to solve visual question-answering tasks in a modular way. The researchers understood that the questions can be decomposed into concepts that reoccur and thought of specially designed neural network architectures that specialize to each of them separately. The modules themselves contain fully connected convolution and attention components as well as non-linear activations that are composed in a different sequence, depending on the sub-task they need to solve. For example, to combine two already-learned concepts, the corresponding module merges the two attentions from the already-computed visual groundings by stacking first, then applying convolution, and lastly passing the result from a non-linearity. The possible inputs and outputs of the modules are also constrained to be either images, attention, or labels in a way that resembles software application programming interfaces (APIs).^101^

To answer a question, the right modules must be chosen and a combined, modular architecture needs to be created. At training time, a separate neural network learns how to select the set of necessary modules and how to connect them with each other so that they jointly learn the necessary representations to answer the question. This can be a recurrent neural network (RNN) that outputs the textual symbolic expression of the optimal module structure. The search over the space of all possible layouts was made even more efficient with the use of RL^102^ and the incorporation of expert policies that were used for pre-training.

The neural modules did not learn disentangled representations of concepts, needed one dedicated module for each concept and at test time did not show robustness to unseen questions. Nevertheless, they are considered interpretable, have modular structure meaning that they can be “pluggable” in several architectures and were an important starting point for later architectures and research directions such as the relation networks (RNs) (see section “relation networks”) and the neuro-symbolic hybrid models described in section “neuro-symbolic methods.” The communication between modules in an interface-like way, by the same means, that programs for the composition of higher-order concepts from simpler ones are a recent improvement that makes them more extendable.^101^

### Neuro-symbolic methods

Neuro-symbolic methods divide their architecture into two parts; one is processing the data and learning the appropriate embeddings, whereas the other learn symbolic representations of the input data. The representation of the image is thereby disentangled from the symbolic execution engine that processes its symbolic representation, thereby enabling different types of images to be executed by it as long as they can conform to the learned representation scheme.

One of the first works, the Neural-Symbolic VQA (NSVQA),^68^ parses the scene with a state-of-the-art image-processing CNN to extract objects as well as their features (color, shape, size, material, and coordinates). The input question is used as input to an LSTM that outputs programming code to process the structural representation extracted from the image by the CNN. Each logical operation and relation ability required by the model has its corresponding programming language module; the set of all those modules is pre-defined by human domain knowledge and is therefore also interpretable. To test generalization, researchers applied the learned model on a dataset containing Minecraft (https://www.minecraft.net/en-us/) scenes where the resulting structural representations were, as expected, longer than the ones that were generated for the CLEVR dataset (see section “CLEVR”). Generalization to images containing natural scenes that were not encountered at training time is not possible with this method.

To make the representation trainable and less rigid, one has to make it learnable by optimization; this is implemented by the Neuro-Symbolic Concept Learner (NSCL).^47^ The main difference is that, after the image is processed by the CNN, neural operators implemented by simple linear layers of neurons learn the concept embeddings. With the use of curriculum learning, the easiest concepts involving shape and color are learned and separated first, and then the neural operators can learn more difficult ones. The optimization is guided by the REINFORCE algorithm^103^ to produce a program that obeys a specially designed DSL and is therefore interpretable. This helped the overall architecture to have greater generalization capabilities that are tested on scenes containing more objects, and non-encountered attribute combinations as well as learning a completely new color (zero-shot learning). An extended version^77^ that performs classification by discriminating if two concepts have a particular meta-concept relation, which, although it needed an enhanced input dataset considering meta-concepts, produced disentangled representations of concepts and was more data-efficient.

The Probabilistic Neuro-Symbolic VQA (P-NSVQA)^65^ is an extension of the NSVQA described above that incorporates the probability of the prediction instead of only the prediction label (after application of a threshold) itself. The logical reasoning program is executed with a joint probability composed of the prediction probability and the detection probability of objects, their attributes, and their relation ns. In a later work,^104^ the principles of NSCL have been applied to 3D scenes composed of point clouds of objects as well as their relations. Multiple-view—and therefore also disentangled—representations are being fused for the generation of the 3D-grounded language that even achieves zero-shot learning in unseen tasks. Note that each object’s point cloud needs its own PointNet++ network^105^ and each pair of objects its own encoder. By not allowing weight sharing, the researchers achieved a separate encoding of the object’s and relation’s features.

Bahdanau et al.^62^ showed that, for the extension dataset CLOSURE of CLEVR (see section “CLEVR”), neuro-symbolic solutions do not generalize well. The user’s domain knowledge is a requirement for the design of this system and, for any new dataset, the adaptation overhead is bigger and non-systematic compared with other technical solutions.

Visual grounding of images and sampled constituency tree representations are learned in alternation with a loss that encourages their alignment. The REINFORCE algorithm^103^ is used as in Mao et al.^47^ for gradient estimation of the parameters; the reward uses the concreteness of the representation of each text constituent and discourages abstract ones that do not have the corresponding grounding. Furthermore, data from other modalities, such as different languages, do help the performance of the algorithm.

### RNs

RNs^106^ consist of a specially designed architecture that has high performance on the Sort-of-CLEVR dataset (see section “CLEVR”) and that focuses on exercising specifically the relational reasoning capabilities. The parameters of the neural network learn relations between the objects in a way that draws parallels with the way CNNs learn weights to capture the translation invariances in the input images or the dependencies between the input sequence in the case of RNNs. Each pair of objects is linearly weighted, consisting of the input to an individual non-linear function; the sum of all of them is, in turn, parameterizable, making the model considerably complex (quadratically) w.r.t. the number of objects in the image. The authors draw parallels on their solution with graph theory since their model operates on a complete graph; in later solutions (see section “GNNs”) of concept learning, RNs are also seen as graph neural networks (GNNs).^107^ The model uses an image-processing part and the CNN feature maps are used as input to the relational network; at the same time, the question is processed by an LSTM that conditions the RN with question embeddings; the relations are learned independently from object recognition. The results indicate that the successful architectures for the solution of those tasks must contain separated components for input structure processing and dedicated modules for relational reasoning.

The performance and robustness of RNs were improved recently by “pluggable” modules called set refiner networks (SRNs). The main idea is based on the acknowledgment that the effectiveness of a neural network depends on the vector representations of the input elements computed at the perceptual stage (which, in the case of images, is usually learned by a CNN). SRN modules consist of a stage between the input embedding and the reasoning component, but, instead of mapping the embedding to a set, they encode the set representation to an input embedding that can be passed onto the RN. Those representations are shown to be decomposed properly and thereby support the relationship learning task, even in cases where an input entity belongs to many set elements. Iterative inference refines an initial output of a set generator to search for its mapping to an appropriate embedding in an unsupervised fashion. Experiments in the image-processing domain, comparing the number of derived set elements and the number of objects, as well as tasks considering translations, measure the effectiveness of this representation. The Sort-of-CLEVR^53^ extension of CLEVR (see section “CLEVR”) is shown to be solvable with higher performance with the use of the SRN module, without any other quantitative changes in the RN architecture. Furthermore, SRNs have shown their value in representation learning through RL of the environment’s state and in textual relations detection where the set encoder uses a GNN to create an iteratively refinable graph vector representation.

### GNNs

Graphs and GNNs are currently used extensively in image segmentation,^108^ text processing,^109^ biological network analysis,^110^ and xAI methods.^111^ More specifically, concept graphs^111^ consist of an attempt to compute a graph from the concepts that are learned by trained neural network models and relations thereof. They draw inspiration from Bayesian models (which are also graphical models^112^^,^^113^) that have interpretable random variables but lack performance and abilities to generalize to group the weights of trained deep neural networks with hierarchical clustering. The ultimate goal is to find active inference trails in the created graphical model based on assumptions about the network weights and create visual trail descriptions that will be validated by biomedical professionals^114^^,^^115^.

Compositional generalization in both CLEVR and CLOSURE datasets (see section “CLEVR”) has also been achieved with high performance using multimodal GNNs.^107^ The caption or question text, as well as the image scene, consist of the two modalities that are represented as graphs; the common GNN (which is a graph isomorphism network [GIN]) calculates the correspondence between them. Joint learning of the representations by fusion is beneficial over the symbolic approaches discussed in section “neuro-symbolic methods” for scaling to more natural images, with more complex objects and longer text as well as joint compositional reasoning. The learned GNN embedding is used for downstream tasks, such as caption truth prediction tasks and generalization tests, not only with the good overall performance but specific to all different concept learning subtasks.

An effective solution for the RPM matrices, as well as the Euler diagram syllogism,^116^ is given by wang et al.^117^ The performance of this solution is better than the previously developed RNs^106^ (described in section “relation networks”), although the ultimate goal of this work is also to capture the relations between the objects of different images. The loss that is minimized is identical to Barrett et al.,^106^ but the methodology uses multiplex graph networks that process relations embeddings. One of the key ideas is that the graph does not have the objects as nodes like a scene graph but uses the summarization of graphs. The overall architecture contains a pipeline of object representation, graph processing, and reasoning; each of them passes the embeddings to the next one. To enable the algorithm to succeed in several different concept learning tasks, different aggregation types are used; concepts that compare size use maximum and minimum feature aggregations, whereas sum is going to be necessary for the counting of objects. It is argued that symbolic methods such as the ones described in section “neuro-symbolic methods” would not be effective in the RAVEN dataset, since this dataset does not provide a question to trigger the creation of a grammar or program. Nevertheless, the need for logical rule extraction from the learned entangled representation is explicitly stated as a requirement for interpretability. Arenas et al.^42^ further argue that more symbolic interpretability tools are needed in order to allow humans to naturally understand and interpret machine learning models and their decisions since humans reason similarly.

Overall, GNNs are a promising direction for further research for concept and representation learning as well as visual question answering, since they provide new desirable properties that previous neural network architectures did not have. For example, neuro-symbolic methods (see section “neuro-symbolic methods”) can be improved with the application of GNNs^118^^,^^119^ by processing the probabilistic scene graph extracted from the image in a different way than the causal models described in section “causal models.” Furthermore, multimodal GNNs can express counterfactuals^120^ that have been shown to be profitable for visual QA solutions,^121^ enhancing particularly the generalization capabilities of the models.^122^

## Future challenges and research directions

### Causal models

Causal generative models are also used to infer the scene-generation process of benchmark datasets per se.^123^ Prior knowledge in the form of assumptions and inductive biases about their dependencies and form is encoded by the pre-defined structure of a probabilistic graphical model (PGM) that uses variational inference as a means to learn its parameters from data. After this unsupervised model is fixed, a competition between the mixture elements is performed and the most likely composition of objects emerges as the result. The use of attention^124^ helps select the image regions containing objects and can deal with occlusion as well as scene depth. Thereby, all possible compositions of objects as well as their attributes and relations are sequentially “explained away” and all recombinations of their representations are supported in the generative phase. The use of such models for explainability—which is considered built-in per design—and concept, as well as representation learning, is a promising research direction.

The work of Hudson and Manning^69^,^125^ over the years is also concentrated on solving the concept learning and reasoning challenges with the use of causal models. Their research focuses on the computation of a neural state machine that extracts a probabilistic scene graph from each input image and expresses thereby the objects, attributes, and relations. The probabilistic model will be able to answer questions by applying inference to the causal model in a sequential fashion. Each question is decomposed into reasoning parts; an inference procedure needs to be applied by traversing the causal variables involved to provide the answer. This model also uses domain knowledge, since the semantic concepts are pre-defined and can be used for the factorization of the model. This is exactly what provides the desired disentanglement properties and the required modularity. The embeddings of objects, attributes, and relations are defined in an initial state, and the goal of the training procedure is to align the degree of belief for each detected component of the image with the corresponding embedding. Since each entity in the image is represented by a set of vectors, the created representations are disentangled and can be recombined at test time on datasets with different distributions, contexts, and text conforming to different grammatical rules with good generalization properties. On the other hand, rarely encountered entities cannot have a good representation, thereby hindering generalization performance. The random variables do provide greater interpretability, since they express known components, but do not support completely unseen configurations that do not have a corresponding random variable properly.

### RL-inspired solutions

The work of Misra et al.^16^ is inspired by design principles of RL^102^ to tackle the concept learning problem of CLEVR dataset (see section “CLEVR”) not by means of a specially designed neural network architecture but by learning to adjust the dataset presented to the model during training. The architecture consists of a typical image-processing neural network and a text-processing one that is conditioned on the extracted image features of the first. Nevertheless, the training procedure does not rely on a passive dataset; the model even learns the language model that the questions should follow through a process called visual question generation (VQG). The overall architecture consists of a question generator that is trained to compute questions that are relevant so that the answering module can learn a policy that is driven by rewarding positively the expected accuracy improvement; this expresses the informativeness value of the question that was selected.

The researchers showed that the concepts occurring during the learning phase proceed from the easiest ones to the most difficult ones, which resembles curriculum learning, which was explicitly used in neuro-symbolic solutions, as described in section “neuro-symbolic methods,” and in this work, it is emerging. Furthermore, this method is more sample efficient, learns autonomously which questions are more profitable to ask long term, and has an actionable behavior on discovering which questions are invalid, redundant, or more difficult than appropriate. The results showed the model, although trained on a dataset that does not have the same distribution in the training and validation set as CLEVR does, has comparable performance with explicitly designed models trained on CLEVR. In the case of CLEVR-Humans, where the distribution is not the same, it has better performance, thereby indicating increased generalization capabilities.

### Cognitive xAI

Cognitive xAI deals with the generation of rule-based explanations.^126^ It can be combined with already-established xAI methods and enhances them by bringing the human in the loop.^114^ Domain experts could, for example, define their own dictionary with content related to cognitive concepts that is independent of the xAI method. Currently, there is a lack of datasets to benchmark trained models for cognitive XAI. If any, there exist benchmarks that are still in creation and not yet sophisticated enough for comprehensive evaluations. One benchmark simulates explanations for decisions of GNNs with the help of validatable rules.^88^ The approach presented there allows for learning human-understandable rules on sub-graphs that have been considered relevant by a GNN. Depending on the learning task and the type of data, the dictionary (concepts and relations between them) has to be tailored to the model or explainers used in combination with rules, Nevertheless, rules that are produced by an interpretable machine learning method, or used as validatable *post hoc* explanations for black box models, enhance the comprehensibility of automated decision making.^127^ This said, there is still a need for relational benchmarks to test the match between human dictionaries and model encoded concepts and the expressiveness and faithfulness of generated rule-based explanations, respectively.

### Conclusions

Benchmarking reasoning abilities, efficient skill acquisition, and learning adaptivity to newly presented data and concepts is an ongoing research direction that draws ideas and knowledge from human and intelligence tests.^22^ The creation of a fair, reproducible, transparent, and compelling intelligence estimation set of tasks requires the balance of prior knowledge, continuous skill acquisition, appropriate difficulty, and clear but not rigid goals. The aim of human developers and data scientists is to accompany newly developed AI solutions toward a path of gradual expansion of their problem-solving horizon by making the steps from local to broad generalization learnable and interactive. New abilities emerge from AI solutions that are exercised to meet the reasoning and generalization abilities of new concept learning benchmark datasets and at the same time their deficiencies, shortcomings, as well as their effectiveness will initiate the search for new cognitive frontiers that need to be reached.
